# TSCL-LwF: A Cross-Subject Emotion Recognition Model via Multi-Scale CNN and Incremental Learning Strategy

**DOI:** 10.3390/brainsci16010084

**Published:** 2026-01-09

**Authors:** Chunting Wan, Xing Tang, Cong Hu, Juan Yang, Shaorong Zhang, Dongyi Chen

**Affiliations:** 1 School of Electronic Engineering and Automation, Postdoctoral Mobile Station for Instrument Science and Technology, Guilin University of Electronic Technology, Guilin 541004, China; wallct@guet.edu.cn (C.W.); hucong@guet.edu.cn (C.H.); 2 School of Electronic Information and Automation, Guilin University of Aerospace Technology, Guilin 541004, China; yangjuan@guat.edu.cn (J.Y.); zsrong@guat.edu.cn (S.Z.); 3 School of Automation Engineering, University of Electronic Science and Technology of China, Chengdu 611731, China; dychen@uestc.edu.cn

**Keywords:** EEG, cross-subject emotion recognition, multi-scale convolutional, incremental learning, Learning without Forgetting (LwF), sparse-channel

## Abstract

**Background/Objectives**: Wearable affective human–computer interaction increasingly relies on sparse-channel EEG signals to ensure comfort and practicality in real-life scenarios. However, the limited information provided by sparse-channel EEG, together with pronounced inter-subject variability, makes reliable cross-subject emotion recognition particularly challenging. **Methods**: To address these challenges, we propose a cross-subject emotion recognition model, termed TSCL-LwF, based on sparse-channel EEG. It combines a multi-scale convolutional network (TSCL) and an incremental learning strategy with Learning without Forgetting (LwF). Specifically, the TSCL is utilized to capture the spatio-temporal characteristics of sparse-channel EEG, which employs diverse receptive fields of convolutional networks to extract and fuse the interaction information within the local prefrontal area. The incremental learning strategy with LwF introduces a limited set of labeled target domain data and incorporates the knowledge distillation loss to retain the source domain knowledge while enabling rapid target domain adaptation. **Results**: Experiments on the DEAP dataset show that the proposed TSCL-LwF achieves accuracy of 77.26% for valence classification and 80.12% for arousal classification. Moreover, it also exhibits superior accuracy when evaluated on the self-collected dataset EPPVR. **Conclusions**: The successful implementation of cross-subject emotion recognition based on a sparse-channel EEG will facilitate the development of wearable EEG technologies with practical applications.

## 1. Introduction

Emotions encompass diverse types of subjective mental experiences, playing an important role in daily life, influencing human decision-making, planning, and other psychological states [1]. With the rapid development in the areas of computer technology, artificial intelligence, wearable technology, and information fusion technology, machines and computers have the ability to understand, recognize, and analyze emotions [2]. Emotion recognition has a broad range of applications, such as human–computer interaction [3], epilepsy detection [4], military and aerospace applications [5], and depression detection [6].

Emotions are usually expressed through physiological responses and behaviors. Compared to behaviors such as facial expressions, vocalizations, and body movements, physiological signals are regarded as offering more precise reflections of a subject’s emotions, as they are beyond the direct control of any subject’s intentions [7]. Among the various physiological signals, electroencephalogram (EEG) measurements are noninvasive, cost-effective, easy to perform, and have a high temporal resolution [8]. Therefore, a growing number of researchers are considering utilizing EEG signals for emotion recognition.

However, traditional EEG measurement devices are primarily designed for medical and research applications. These devices are generally bulky, cumbersome, and operationally demanding, which limits their applicability for daily use in real life [9]. To address these shortcomings, many wearable EEG devices based on dry electrodes are being adopted in research on emotion recognition. For example, Diaz et al. [10] acquired eight-channel EEG signals and motor data to analyze the emotional state of subjects while playing a whack-a-mole game. Ma et al. [11] presented a novel portable EEG signal acquisition and analysis system with a 10-channel dry electrode device. The system is supported by a channel selection network based on Squeeze-and-Excitation (SE) attention and multi-scale convolution to enhance classification performance. These studies demonstrate that with the advancement of wearable technology, the potential of emotion recognition in natural settings using dry-electrode wearable EEG devices is increasingly being realized.

Designing effective discriminant models, which may include deep learning and machine learning techniques, is crucial for EEG-based emotion recognition [12]. Deep learning models can not only further learn based on manually extracted features, but also automatically abstract characteristics from raw EEG signals and train the discriminator at the same time. When dealing with emotion classification tasks, deep learning models can effectively mine the deep feature representation of EEG data and are widely used due to their excellent performance [13].

Deep learning models, such as Convolutional Neural Networks (CNNs), are capable of learning localized feature representations and achieve promising results in EEG classification tasks. For example, Song et al. [14] developed a dynamic graph convolutional neural network (DGCNN). This network effectively extracts the cross-channel interactions in EEG signals. Cheng et al. [15] proposed MSDCGTNet, which extracts the spectral, spatial, and temporal information of EEG signals through CNNs and a gated transformer encoder. Jin et al. [16] constructed a novel network called the pyramidal graph convolutional network (PGCN), which can integrate features at local, mesoscopic, and global levels, while analyzing structural and functional connectivity at each level. Jiménez et al. [17] presented IL2FS, which incorporated weight alignment, margin ranking loss, and triplet loss to maintain inter-class discriminability and feature space alignment for known classes. Han et al. [18] presented a new model combining a multi-scale convolution and a TimesNet network. This model employs various convolutional kernels to capture dynamic spatio-temporal relationships, and subsequently TimesNet is utilized to build 2D sequences and learn intricate temporal features. Tang et al. [19] designed an emotion recognition framework based on an Efficient Capsule Network with Convolutional Attention (ECNCA). They initially concatenated and fused features from specific frequency bands of EEG signals, then used ECNCA to augment the input data with CNN and attention mechanisms, and finally applied Efficient Capsule to categorize the emotions.

**Figure 1:**
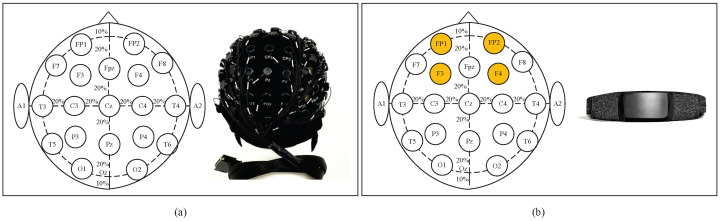
(**a**) Multi-channel EEG system: Electrode layout and matching EEG device. (**b**) Sparse-channel (4-channel) EEG system: Electrode layout and matching EEG device.

Nevertheless, these methods rely on more EEG channels, which means reduced comfort and higher hardware costs. In addition, using multi-channel EEG signals complicates the processing algorithms, constraining their applications for real-time scenarios, particularly in environments requiring low-latency responses. Thus, it is necessary to develop emotion recognition methods based on sparse-channel EEGs for applications in natural environments. As illustrated in Figure 1, reducing the number of EEG channels significantly facilitates signal acquisition using wearable devices, improving comfort and practicality in real-world applications. Based on the left–right brain asymmetry theory, the sparse channels employed in this study are selected from the left and right hemispheres (i.e., FP1, FP2, F3, and F4). All the electrodes corresponding to these channels are located in the prefrontal cortex (in accordance with the international 10–20 system), unobstructed by hair, and have been shown to be closely related to emotions.

Cross-subject emotion recognition is emerging as a future trend due to its ubiquity and wider applications [20]. Shi et al. [21] proposed an EEG cognitive recognition method based on a spiking neural network, in which a sample-based adaptive thresholding strategy was introduced to improve generalization ability. Li et al. [22] put forward a cross-subject emotion training method based on a spatial–temporal graph attention network, which could capture time–frequency information and intrinsic relationships between different EEG channels to achieve higher classification accuracy. Li et al. [23] presented the fusion model of a multi-scale residual network (MSRN) and a meta-transfer learning (MTL) strategy. The MTL strategy combines the advantages of meta-learning and transfer learning to reduce individual differences among subjects. Shen et al. [24] constructed a network called CLISA based on contrastive learning with a training phase incorporating a specially formulated loss function. The network combines temporal and spatial convolution and eventually obtained 47% accuracy on the THU-EP dataset. Ding et al. [25] developed a new transformer method called emotion transformer (EmT) which consisted of a residual multi-view pyramid GCN module and a temporal contextual transformer. The method attained an accuracy of 59.5% on the THU-EP dataset. In addition, domain-adaptive methods in transfer learning can effectively eliminate inter-subject neural representation differences. These methods achieve robust decoding of cross-subject emotional states. For example, Quan et al. [26] introduced a new feature extraction method called MR-VAE, as well as a multi-source domain transfer learning model for selecting the optimal transferable samples from both global and subdomain distributions. Ni et al. [27] proposed CTDDSR, which combines migration learning, dictionary learning, and linear discriminant analysis techniques. The model enables different domains to utilize a shared dictionary, extract details from the EEG signals of current domains, and transfer the knowledge to a new domain, which effectively minimizes labeled data demand for target subjects and alleviates the differences between the source and target domains.

Existing domain adaptation methods typically require explicit source–target alignment and simultaneous access to both domains, which is impractical for wearable EEG applications. On the other hand, such methods are sensitive to severe inter-subject variability and sparse-channel constraints. Furthermore, incremental learning approaches have mainly focused on class-incremental scenarios rather than addressing the subject differences in cross-subject emotion recognition. In addition, most existing incremental learning frameworks are not designed to work with feature extraction architectures tailored to sparse-channel EEG signals.

To mitigate the mentioned limitations, we propose a cross-subject emotion recognition model (TSCL-LwF) based on a sparse-channel EEG, which combines a multi-scale convolutional network (TSCL) and an incremental learning strategy with LwF. Specifically, in the TSCL model, a multi-scale architecture is used to capture the temporal dynamics and inter-channel spatial correlations of the local prefrontal region (with all sparse-channel readings confined to the prefrontal area), while its separable convolutional layers model inter-channel dependencies by fusing multi-level features extracted from the sparse-channel EEG signals. The incremental learning strategy with LwF introduces a limited set of labeled target domain data to enable the TSCL-LwF model to rapidly adapt to the target domain data distribution. Meanwhile, LwF leverages the knowledge distillation loss to retain prior knowledge learned from source subjects to reduce the differences between subjects, thus enhancing the generalization ability and recognition accuracy of cross-subject emotion recognition.

The contributions of this paper are as follows:

1. A new multi-scale convolutional model called TSCL is proposed, which utilizes a multi-scale architecture and separable convolutional layers to ensure spatio-temporal feature extraction and interaction, rendering it especially effective for capturing domain-invariant characteristics from sparse-channel EEG signals.
2. An incremental learning strategy with LwF is introduced. It utilizes a limited set of labeled target domain data to enable TSCL-LwF to rapidly adapt to the target domain, while leveraging knowledge distillation loss to reduce the differences between subjects. This strategy enhances the performance of cross-subject emotion recognition.
3. Extensive experiments on the DEAP and EPPVR datasets verified the effectiveness of TSCL-LwF, and it obtained better performance for valence, arousal, and valence–arousal classification with a sparse-channel EEG compared to other methods.

The rest of this article is organized as follows: Section 2 introduces the proposed TSCL-LwF. Section 3 presents the datasets and experimental details. Section 4 elaborates the experimental results and analysis. A discussion of the results is given in Section 5, and Section 6 offers the conclusion of the paper.

## 2. Methods

### 2.1. Overview of TSCL-LwF

**Figure 2:**
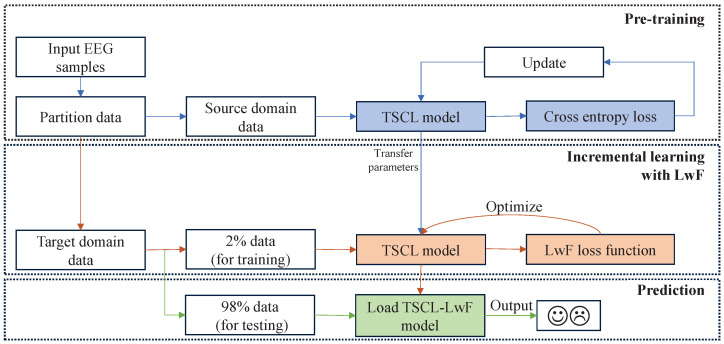
Illustration of the proposed TSCL-LwF model.

As illustrated in Figure 2, the proposed TSCL-LwF model consists of three phases: pre-training, incremental learning with LwF, and prediction. First, the input EEG samples are partitioned into source domain data and target domain data. Next, in the pre-training phase, the source domain data is fed to the TSCL model to extract and fuse spatio-temporal interaction features of sparse-channel EEG signals and generate corresponding prediction labels. Then, the cross-entropy loss is calculated to update the parameters of the TSCL model. After pre-training, the basic TSCL model proceeds to the incremental learning with LwF phase to adapt to the target domain. During this phase, 2% of labeled target domain data is fed to the pre-trained TSCL model, which is fine-tuned using the LwF approach to align the data distributions of the source and target domains and achieve fast convergence to the target domain data. Finally, in the prediction phase, the fine-tuned TSCL-LwF model is directly utilized, and the remaining 98% of the target domain data is employed for emotion classification. In the following subsections, the details of the proposed TSCL-LwF are given.

### 2.2. TSCL Structure

**Figure 3:**
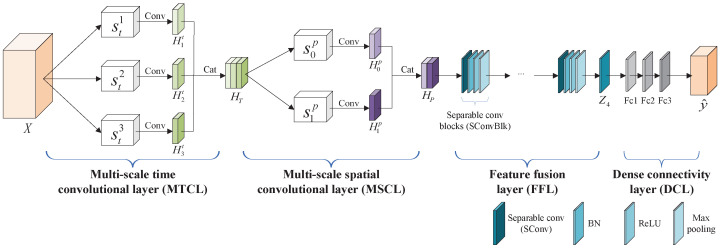
The structure of the TSCL.

The structure of the TSCL is shown in Figure 3, comprising four layers: a multi-scale temporal convolutional layer (MTCL), a multi-scale spatial convolutional layer (MSCL), a feature fusion layer (FFL), and a dense connectivity layer (DCL). Specifically, as EEG signals contain abundant temporal information, sequence modeling is particularly appropriate for processing such time series data. The MTCL is designed to extract dynamic temporal characteristics, capturing fine-grained transient changes and coarse-grained stable patterns in time sequences. The MSCL aims to capture cross-channel spatial information, identifying both local channel-wise interactions and global spatial distributions. From a representation learning perspective, the combination of the MTCL and MSCL enables the TSCL to jointly learn multi-resolution temporal patterns and inter-channel spatial dependencies from sparse-channel EEG data, forming structured spatio-temporal feature representations. The FFL employs separable convolutional layers to fuse the spatio-temporal features extracted from sparse-channel EEG signals and to make the TSCL more compact and efficient. The DCL is employed to complete the final emotion classification.

In the MTCL, three different sizes of convolution kernels are introduced to extract temporal information at different scales. Let *X* be a sequence of temporal sample of sparse-channel EEG signals, denoted as $X=\left[X_{0},X_{1},\ldots ,X_{N}\right],X_{N}\in R^{c*f}$, where *N* represents the number of samples, *c* represents the number of channels, and *f* represents the temporal length of each sample. In order to capture the receptive fields of different scales from the input samples and extract the temporal features corresponding to these scales, we use three different sizes of convolution kernels, denoted as $s_{t}^{i}=\left(1,m^{i}\cdot f\right),i\in \left\{1,2,3\right\},m=0.5$. The output of the $i_{th}$ convolutional layer in the MTCL is represented as $H_{i}^{t}$, which is calculated by Equation (1):

(1) $$H_{i}^{t}=F_{\mathrm{AP}}\left(\mathrm{ReLU}\left(\mathrm{Conv}\left(X,s_{t}^{i}\right)\right)\right),\;i\in \left\{1,2,3\right\},$$

where *X* represents the input samples, $s_{t}^{i}$ represents the convolution kernel size, Conv(·) represents the convolution operation, ReLU represents the activation function, and $F_{\mathrm{AP}}\left(\cdot \right)$ represents the average pooling operation. Next, each output map is concatenated across the feature dimension. To address the internal covariate shift issue within the neural network, a batch normalization (BN) layer is introduced following each concatenation. The final output can be calculated by Equation (2):

(2) $$H_{T}=F_{\mathrm{BN}}\left(\left[H_{1}^{t},\ldots ,H_{i}^{t}\right]\right),\;i\in \left\{1,2,3\right\},$$

where $F_{\mathrm{BN}}\left(\cdot \right)$ represents the batch normalization operation and [.] is the concatenation operation of the output of the $i_{th}$ convolutional layer across the feature dimension.

In the MSCL, two different sizes of convolution kernels are utilized to learn spatial information: global and local kernels. These kernels are denoted as $s_{j}^{p}=\left(m^{j}\cdot c,1\right),j\in 0,\;1,\;m=0.5$, where *c* represents the number of channels. The global kernel $s_{0}^{p}$ matches the channel dimension to capture global spatial characteristics, while the smaller local kernel $s_{1}^{p}$ focuses on cross-channel spatial information. The output of the $j_{th}$ convolutional layer in the MSCL is denoted as $H_{j}^{p}$, which is calculated using the following formula:

(3) $$H_{j}^{p}=F_{\mathrm{AP}}\left(\mathrm{ReLU}\left(\mathrm{Conv}\left(H_{T},s_{j}^{p}\right)\right)\right),\;j\in \left\{0,1\right\},$$

where $H_{T}$ represents the output of the MTCL, $s_{j}^{p}$ represents the convolution kernel size, Conv(·) represents the convolution operation, ReLU represents the activation function, and $F_{\mathrm{AP}}\left(\cdot \right)$ represents the average pooling operation. Similar to the MTCL, each output is concatenated and normalized. The final output is shown in Equation (4):

(4) $$H_{P}=F_{\mathrm{BN}}\left(\left[H_{0}^{p},\ldots ,H_{j}^{p}\right]\right),\;j\in \left\{0,1\right\},$$

where $F_{\mathrm{BN}}\left(\cdot \right)$ represents the batch normalization operation and [.] is the concatenation operation of the output of the $j_{th}$ convolutional layer across the feature dimension.

The FFL is used to integrate the extracted spatio-temporal interaction features. It consists of a stack of separable convolutional blocks (SConvBlk), each containing four layers: a separable convolutional layer (SConv), a BN layer, a ReLU layer, and a max pooling layer. Each SConv includes a depthwise convolution and a pointwise convolution. The depthwise convolution uses a convolution kernel of size $C_{in}\times k\times k$ to perform convolution along the spatial dimensions on the input features, with the number of kernel channels matching that of the input features. The pointwise convolution employs a 1×1 convolution kernel to linearly merge the outputs from the depthwise convolution operation, thereby fusing inter-channel information. The computation of the first SConvBlk can be simplified by the equation $Z_{1}=\mathrm{Reshape}\left(\delta (\mathrm{SConv}\left(H_{P}\right)\right))$, where $H_{P}$ represents the output of the MSCL, SConv(·) represents a separable convolution operation, δ(·) represents the composite operation incorporating BN layer, ReLU layer, and max pooling layer (applied sequentially after each SConv), and Reshape(·) represents the reshape operation. The $q_{th}$ SConvBlk is computed similarly to the first one by Equation (5):

(5) $$Z_{q}=\left(\delta (\mathrm{SConv}\left(Z_{q-1}\right))\right),\;q\in \left\{2,3,4\right\}.$$

The DCL consists of three fully connected layers (fc layers). The output from the FFL is fed to these layers to generate the final emotion classification probabilities. The definition is as follows:

(6) $$\hat{y}=\mathrm{softmax}\left(\mathrm{Linear}\left(Z_{4}\right)\right),$$

where $Z_{4}$ represents the output of the FFL, softmax(·) represents the activation function, and Linear(·) represents the fully connected layers.

### 2.3. Incremental Learning with LwF

Incremental learning enables models to not only learn new knowledge from new samples but also retain most previously learned information. When updating the network, only the parts related to new data require fine-tuning and the model does not need full retraining. Among regularization-based incremental learning methods, the LwF method proposed by Li et al. [28] is widely used. This method constructs a distillation loss to strengthen the correlation between current task knowledge and previously learned knowledge, thus effectively addressing the problem of catastrophic forgetting.

**Figure 4:**
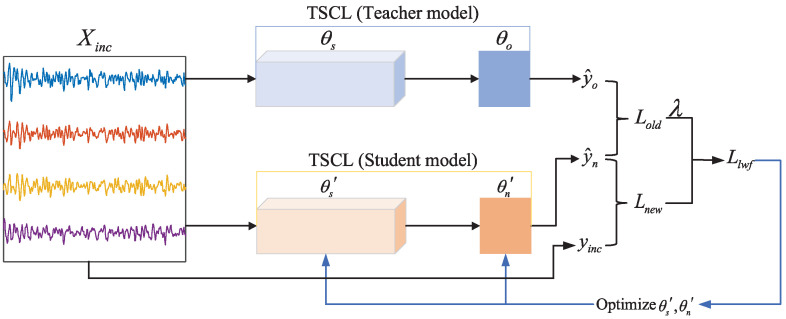
The training process of LwF.

Due to the limited annotated data of new subjects, conventional fine-tuning of pre-trained models can easily lead to overfitting to noisy features or personalized irrelevant features of new subjects. In this work, a limited set of labeled target domain data ($X_{inc}$) is utilized as the incremental dataset, and the pre-trained TSCL model is fine-tuned using LwF. This enables the model to rapidly adapt to the target domain data and further align the data distributions between the source and target domains, thereby improving recognition accuracy. The training process of LwF is illustrated in Figure 4. First, the samples $X_{inc}$ are fed into both the student and teacher models. Subsequently, the LwF loss $L_{lwf}$ is computed based on the output discrepancy between these two models, and this loss is then used to optimize the parameters of the student model. In this process, the pre-trained TSCL model serves as the teacher model, and the student model inherits the teacher model’s parameters and simultaneously initializes the DCL parameters ${\theta_{n}}^{\prime }$. Notably, the teacher model does not participate in backpropagation during this process.

The LwF loss $L_{lwf}$ consists of regular loss $L_{new}$ and distillation loss $L_{old}$, which is defined below:    

(7) $$\begin{array}{c} L_{lwf}=L_{new}\left({\hat{y}}_{t},\;y_{inc}\right)+\lambda L_{old}\left({\hat{y}}_{o},\;{\hat{y}}_{n}\right),\\ {\hat{y}}_{n}=M\left(X_{inc},\;{\theta_{s}}^{\prime },\;{\theta_{n}}^{\prime }\right),\;{\hat{y}}_{o}=M\left(X_{inc},\;\theta_{s},\theta_{o}\right),\; \end{array}$$

where M represents the pre-trained TSCL model, $X_{inc}$ denotes a limited set of labeled target domain data, $y_{inc}$ corresponds to the actual label, ${\hat{y}}_{n}$ and ${\hat{y}}_{o}$ are, respectively, the outputs of the student and teacher models, λ is the relaxation parameter, $\theta_{o}$ and ${\theta_{n}}^{\prime }$ are, respectively, the parameter sets of the DCL from the teacher model and student model, and $\theta_{s}$ and ${\theta_{s}}^{\prime }$ are, respectively, the parameter sets of the layers before the DCL from the teacher model and student model.

The regular loss $L_{new}$ assesses the accuracy of sample classification through common cross-entropy loss. The relevant calculation formulas are expressed below:

(8) $$L_{new}\left({\hat{y}}_{n},\;y_{inc}\right)=-\frac{1}{N_{inc}}\sum_{i=1}^{N_{inc}}y_{inc,i}\cdot \log{\hat{y}}_{n,i},$$

where $N_{inc}$ denotes the number of samples in the labeled target domain data, $y_{inc,i}$ represents the actual label of the $i_{th}$ sample, and ${\hat{y}}_{n,i}$ represents the predicted probability vector of the $i_{th}$ sample output by the student model. The distillation loss $L_{old}$ utilizes Kullback–Leibler (KL) divergence to assess the degree of approximation between the probability distributions output by the teacher model and the student model. Through minimizing the KL divergence, the teacher model can effectively transfer its learned knowledge to the student model. This guarantees that the output of the student model closely aligns with that of the teacher model. It is given in the following definition:

(9) $$\begin{array}{cc} L_{old}\left({\hat{y}}_{o},\;{\hat{y}}_{n}\right) & =-\frac{1}{N_{inc}}\sum_{i=1}^{N_{inc}}\sum_{c=1}^{c}{\hat{y}}_{o,i,c}\log{\hat{y}}_{n,i,c},\\ {\hat{y}}_{o,i,c} & =\frac{{\left({\hat{y}}_{o,i,c}\right)}^{1/T}}{\sum_{c^{\prime }=1}^{c}{\left({\hat{y}}_{o,i,c^{\prime }}\right)}^{1/T}},\;{\hat{y}}_{n,i,c}=\frac{{\left({\hat{y}}_{n,i,c}\right)}^{1/T}}{\sum_{c^{\prime }=1}^{c}{\left({\hat{y}}_{n,i,c^{\prime }}\right)}^{1/T}} \end{array}$$

where *c* denotes the number of classes in the classification task and *T* denotes the temperature coefficient.

### 2.4. Training Process

The entire training process of the proposed TSCL-LwF model is detailed in Algorithm 1, and the method of this work can be summarized as follows:

- *Step 1*: Train the TSCL model on the source domain data and save the converged model as the teacher model.
- *Step 2*: Initialize a student model that inherits the base parameters from the teacher model and randomly initialize new parameters for the DCL module. Then, select 2% of labeled target domain data as the incremental dataset, and feed it into both the teacher and student models. Subsequently, optimize the student model parameters by computing gradients based on the loss function defined in Equation (7), while keeping the teacher model frozen (i.e., no backpropagation).
- *Step 3*: Use the fine-tuned TSCL-LwF model to classify the remaining 98% of the target domain data.

**Algorithm 1** The training process of TSCL-LwF.**Require**: Source domain data $X_{s}$, target domain data $X_{t}$, source domain labels $y_{s}$, target domain labels $y_{t}$, number of pre-training epochs $N_{s}$, number of incremental learning epochs $N_{t}$  1:**Step 1: Pre-training phase**  2:Randomly initialize TSCL model parameters θ  3:**for** epoch=1 **to **$N_{s}$ **do**  4:   Extract MTCL feature vectors $H_{T}$ from $X_{s}$ (Equations (1) and (2))  5:   Extract MSCL feature vectors $H_{P}$ from $H_{T}$ (Equations (3) and (4))  6:   Compute $Z_{4}$ with $H_{P}$ as input (Equation (5))  7:   Obtain predicted labels ${\hat{y}}_{s}$ for source domain (Equation (6))  8:   Calculate cross-entropy loss: $L_{CE}=-\frac{1}{|X_{s}|}\sum y_{s}\cdot \log{\hat{y}}_{s}$  9:   Update model parameters θ via Adam optimizer10:**end for**11:**Save pre-trained TSCL parameters:**$\;\theta_{pre}=\theta$12:**Step 2: Incremental learning with LwF phase**13:Load $\theta_{pre}\left(including\;\theta_{s},\theta_{o}\right)$ as teacher model (frozen, no backpropagation)14:Initialize student model $\theta^{\prime }\left(including\;\theta_{s}^{\prime },\theta_{n}^{\prime }\right)$:15:   Inherit $\theta_{pre}$ as base parameters $\theta^{\prime }$16:   Randomly initialize DCL module parameters $\theta_{n}^{\prime }$17:Extract 2% labeled target domain data as incremental dataset: $X_{inc}\subset X_{t}$, $y_{inc}\subset y_{t}$18:**for** epoch=1 **to **$N_{t}$ **do**19:   Teacher model prediction: ${\hat{y}}_{o}=TSCL\left(X_{inc},\theta_{pre}\right)$20:   Student model prediction: ${\hat{y}}_{n}=TSCL\left(X_{inc},\theta^{\prime }\right)$21:   Compute total LwF loss: $L_{lwf}=L_{new}+\lambda L_{old}$ (Equations (7)–(9))22:   Update student parameters $\theta^{\prime }$ via Adam optimizer23:**end for**24:**Save fine-tuned parameters: **$\theta_{fine}=\theta^{\prime }$25:**Step 3: Prediction phase**26:Extract remaining target domain data as test set: $X_{test}=X_{t}\setminus X_{inc}$27:Load TSCL-LwF model with $\theta_{fine}$28:Predict test labels: ${\hat{y}}_{pre}=TSCL-LwF\left(X_{test},\theta_{fine}\right)$29:**return** Predicted labels ${\hat{y}}_{pre}$ for target domain data

## 3. Experiments

### 3.1. Datasets

In order to validate the performance of the proposed model, we used two physiological–emotional analysis datasets: the publicly available DEAP dataset [29] and the self-collected EPPVR dataset [30]. There are significant differences between the two datasets in terms of EEG equipment, stimuli, participant ages, and culture, which enable a comprehensive evaluation of the robustness of the proposed method. Table 1 presents relevant details about both datasets. The DEAP dataset includes 32 subjects, composed of 16 males and 16 females. The ages of the subjects vary from 19 to 37 years, with a mean age of 26.9 years. All 32 subjects watched 40 1-min videos, during which 32-channel EEG signals were recorded. Each EEG recording lasted for 63 s, with the initial 3 s serving as the baseline. After finishing each trial, subjects were requested to assess their emotions on a scale from 1 to 9 regarding arousal and valence dimensions.

The EPPVR dataset records multimodal physiological signals from 30 subjects while viewing 14 VR scenes. Specifically, 30 undergraduate or graduate students were recruited, with ages between 20 and 26 years (with a mean age of 23 years and a variance of 2.3 years), including 18 males and 12 females. Each subject viewed 14 VR scenes. Each VR scene lasted 70 s, of which the first 10 s were baseline signals. For each emotionally triggered trial, 32-channel EEG signals were recorded simultaneously using Ant Neuro’s EEG amplifier ${\mathrm{eego}}^{TM}$ mylab (Ant Neuro b.v., Hengelo, The Netherlands). After finishing each trial, subjects were requested to provide emotional annotations for valence and arousal dimensions via the emotional Self-Assessment Manikin (SAM) scale.

### 3.2. Preprocessing

**Figure 5:**
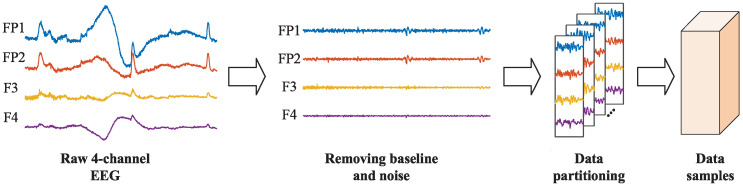
Schematic diagram of the preprocessing steps.

The schematic diagram of the preprocessing steps for EEG signals is shown in Figure 5. Firstly, the 3 s baseline signals were eliminated from each trial. Then, the data was downsampled to 128 Hz, and a 4–45 Hz bandpass filter was applied to remove noise. The filtered data were subsequently re-referenced. Finally, electrooculography (EOG) was eliminated, employing the method outlined in the literature [29]. These preprocessing steps were provided as part of the official DEAP dataset, and the preprocessed EEG signals were directly used in our experiments. To address the requirement for a sparse-channel EEG in natural environment applications, based on the left–right brain asymmetry theory, we selected four EEG channels (i.e., FP1, FP2, F3, F4) from the prefrontal region [31]. Meanwhile, in order to solve the problem of the limited number of samples, we adopted a non-overlapping sliding window of 1 s for data segmentation. Additionally, the EEG signals of each channel were normalized to the range of [0, 1]. After preprocessing, there were 40 × 60 = 2400 data samples per subject (40 trials, each containing 60 1 s signals), totaling 76,800 data samples. Each data sample had a size of 4 × 128 (number of channels of 4 and temporal length of 128). According to previous work [32], in the valence and arousal classification, labels below 5 were classified as low categories, while labels above 5 were classified as high categories. In the valence–arousal classification, labels were classified into four categories: LVLA (valence < 5, arousal < 5), LVHA (valence < 5, arousal ≥ 5), HVLA (valence ≥ 5, arousal < 5), and HVHA (valence ≥ 5, arousal ≥ 5).

For the EPPVR dataset, firstly, the 4-channel EEG signals (FP1, FP2, F3, F4) were downsampled to 100 Hz. Then, the 10 s baseline signals were removed from each trial. Subsequently, the interfering signals outside the frequency range were filtered out utilizing a 4th-order Butterworth bandpass filter (4–45 Hz). Next, each sample was further split into non-overlapping segments of 1 s. Finally, the EEG signals of each channel were normalized to the range of [0, 1] to mitigate the influence of fundamental frequency rhythms on inter-individual and inter-channel variations. After preprocessing, there were 14 × 60 = 840 data samples per subject (14 trials, each containing 60 1 s signals), leading to a total of 25,200 data samples. Each data sample was represented as a matrix of size 4 × 100, corresponding to 4 EEG channels and a temporal length of 100 time points. The labels were preprocessed in the same way as the DEAP dataset.

### 3.3. Implementation Details

We utilized Python 3.8 and the PyTorch 1.8.0 framework to build the proposed TSCL-LwF model, and conducted training on an NVIDIA GeForce RTX 3090 GPU. The TSCL-LwF was trained using optimized parameters: the Adam optimizer was adopted, with a batch size of 128, initial learning rate of 0.001, and dropout of 0.5 to prevent overfitting [33]. To guarantee sufficient EEG feature learning, the numbers of pre-training epochs and incremental epochs were set to 100 and 15, respectively. The random seed was uniformly set to 42 to ensure the consistency and reproducibility of experimental results. The relaxation parameter λ was set to 0.1, which prevented the model from being overly biased towards source domain knowledge due to an excessively high weight. The temperature coefficient *T* was used to regulate the smoothness of the soft labels in knowledge distillation. To better adapt to the target domain, a relatively small coefficient value of T=2 was set. The key parameter settings and architectural details of the model are shown in Table 2 and Table 3. To demonstrate the robustness of the proposed model, we used a consistent set of parameters for experiments on both datasets.

### 3.4. Experimental Setup

To assess the performance of the proposed TSCL-LwF model on two datasets, a leave-one-out cross-validation method was employed [34,35]. Specifically, in each experiment, given *M* subjects in the dataset, the EEG data of M−1 subjects was selected as the source domain data, while the one remaining subject provided the target domain data. Moreover, 2% of the target domain data was allocated as an incremental dataset, which was randomly selected with a fixed random seed (42) to ensure reproducibility, and the remaining 98% of the target domain data was utilized as testing data. Each experiment was repeated 5 times, and the mean performance (±standard deviation, STD) is reported.

### 3.5. Evaluation Metrics

In this work, accuracy and F-Score were adopted as evaluation metrics for emotion classification performance assessment. Accuracy is defined below:

(10) $$\mathrm{Accuracy}=\frac{\mathrm{TP}+\mathrm{TN}}{\mathrm{TP}+\mathrm{FP}+\mathrm{TN}+\mathrm{FN}},$$

where TP and TN, respectively, denote true positive cases and true negative cases, and FP and FN, respectively, denote false positive cases and false negative cases. The formula used to calculate the F-Score is shown below:

(11) $$\mathrm{F-Score}=2\times \frac{\mathrm{Pre}\times \mathrm{Rec}}{\mathrm{Pre}+\mathrm{Rec}}=\frac{\mathrm{TP}}{\mathrm{TP}+(\mathrm{FP}+\mathrm{FN})/2},$$

where Pre denotes precision and Rec denotes recall.

Moreover, the confusion matrix is presented to clearly display the misclassification of each category. By analyzing the confusion matrix, we can attain a better comprehension of how the model performs in classifying different categories.

## 4. Results

### 4.1. Emotion Recognition Performance

**Figure 6:**
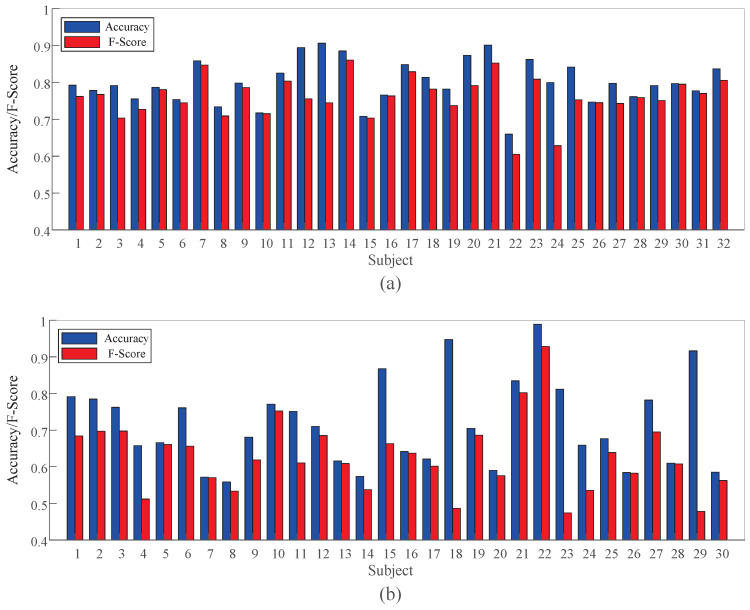
Experimental results of TSCL-LwF for each subject in arousal classification on different datasets. (**a**) DEAP dataset. (**b**) EPPVR dataset.

The accuracy and F-Score of TSCL-LwF for each subject in arousal classification on both DEAP and EPPVR are shown in Figure 6. On the DEAP dataset, the proposed TSCL-LwF achieves an average accuracy of 80.12%. Subjects 7, 12, 14, 20, 21, and 23 show higher accuracy. Subject 21 performs best at 90.14%, while subject 4 has the lowest accuracy at 65.95%. On EPPVR, the average accuracy is 71.62%. Subject 22 achieves the highest result at 98.80%, while subject 8 performs worst at 51.93%. The average F-Scores on DEAP and EPPVR are 76.02% and 62.59%, respectively. Some subjects have lower classification accuracy, which may be due to significant differences in data distribution between these subjects and others. Compared to EPPVR, classification accuracy and F-Score are more stable on DEAP. This suggests that subjects on DEAP have smaller differences than those on EPPVR. The results indicate that although TSCL LwF exhibits differences in classification performance among different subjects, it still achieves competitive average accuracy and F-Score on both datasets.

**Figure 7:**
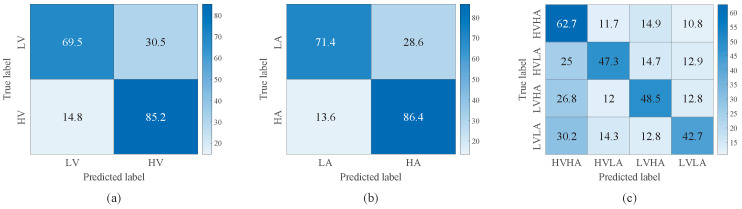
Confusion matrix of TSCL-LwF for emotion classification on the DEAP dataset. (**a**) Valence. (**b**) Arousal. (**c**) Valence–Arousal.

**Figure 8:**
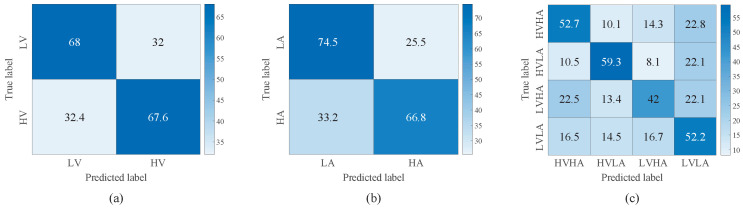
Confusion matrix of TSCL-LwF for emotion classification on the EPPVR dataset. (**a**) Valence. (**b**) Arousal. (**c**) Valence–Arousal.

Figure 7 and Figure 8 illustrate the confusion matrices of TSCL-LwF for emotion classification on the DEAP and EPPVR datasets, respectively. The vertical axis of the confusion matrix denotes the actual emotion labels. The horizontal axis indicates the predicted emotion labels. The results from Figure 7a,b show that for the DEAP dataset, the high categories are easier to distinguish than the low categories, and the accuracy of high categories is at least 15% higher than that of low categories. For the EPPVR dataset (see Figure 8a,b), low categories are easier to distinguish than high categories. The differences may be due to the uneven distribution of samples on the two datasets. On the DEAP dataset, high categories are more prevalent, while on the EPPVR dataset, low categories have a greater number of samples. As shown in Figure 7c, in the valence–arousal classification of DEAP, HVHA emotions are more easily recognized than HVLA, LVHA, and LVLA emotions. According to Figure 8c, in the valence–arousal classification of EPPVR, HVLA classification has an accuracy of 59.3%, while LVHA classification has the lowest accuracy of 42%. Furthermore, 22.5% of LVHA emotions are misclassified as HVHA; this may be due to an unclear boundary between LVHA and HVHA.

### 4.2. Comparison Experiments

To evaluate the performance of the proposed TSCL-LwF, a comparison with existing emotion recognition models was conducted. The comparison models used in the experiments include ShallowConvNet [36], DGCNN [14], CLISA [24], TSception [37], LGGNet [38], MACTN [39], and EmT [25]. All compared baseline models, as well as the proposed TSCL-LwF model, were evaluated under the same sparse-channel EEG setting. Table 4 shows the average accuracy and F-Score obtained on the DEAP dataset, along with the standard deviation. TSCL-LwF achieves an accuracy of 77.26% for valence, 80.12% for arousal, and 51.87% for valence–arousal classification, with corresponding F-Scores of 76.25%, 80.12%, and 42.02%, respectively. Compared with baseline models, TSCL-LwF achieves the best recognition results, and all its performance advantages over baseline models are statistically significant (p< 0.001). In binary classification, TSCL-LwF consistently outperforms these models by at least 20% in terms of accuracy and F-Score. In valence–arousal classification, TSCL-LwF shows a remarkable accuracy improvement of over 22.91% relative to EmT, which is the best supervised-learning-based model in our comparative experiments and employs a residual multi-view pyramid network. Additionally, the accuracy of TSCL-LwF is 11.86% higher than CLISA, which adopts contrastive learning to reduce inter-subject differences. In summary, TSCL-LwF can effectively extract complex spatio-temporal characteristics from sparse-channel EEG signals and retain information with similar features to new subjects, thus improving generalization ability and the accuracy of cross-subject classification.

The comprehensive experimental results on the EPPVR dataset are presented in Table 5. The proposed TSCL-LwF model achieves 70.93%, 67.88%, and 50.63% accuracy in arousal, valence and valence–arousal classification, respectively. In valence classification, TSCL-LwF did not exhibit statistically significant superiority over some comparative methods (e.g., CLISA), which may be attributed to a combination of their narrower performance gap relative to TSCL-LwF and their relatively larger standard deviations. Comparing the results in Table 4 and Table 5, it becomes clear that multi-classification emotion recognition is more challenging than binary classification. Promisingly, TSCL-LwF shows significant superiority in accuracy and maintains a lower standard deviation than other models. This comparison indicates that it has better analytical ability in various tasks and stimulus environments.

## 5. Discussion

### 5.1. Ablation Experiments

In this section, we assess the importance and contribution of each component in the TSCL-LwF model through ablation experiments. We analyze the effects of the MTCL, the MSCL, the FFL, and the LwF method on the emotion classification results. Table 6 shows the results for arousal classification on DEAP and EPPVR. ‘w/o’ means a component is removed. In the ablation experiments, removing the MTCL reduced accuracy by 1.47% (DEAP) and 2.19% (EPPVR) compared to the full TSCL-LwF model. Similarly, omitting the MSCL led to larger accuracy reductions of 12.24% (DEAP) and 8.64% (EPPVR), while excluding the FFL caused drops of 5.05% (DEAP) and 11.36% (EPPVR). These results confirm that the TSCL effectively extracts domain-invariant features from sparse-channel EEG signals for emotion recognition. When LwF is removed, the accuracy decreased by 5.89% and 9.81%, and the F-Score decreased by 4.46% and 5.49%, respectively. This illustrates that LwF is capable of learning the feature information of a new subject while preserving the domain-invariant features. Therefore, integrating the LwF method in the incremental learning stage can improve the effectiveness of the model. The above experiments also prove that omitting any single component reduces the overall performance of the model, with the MSCL showing the most substantial impact on DEAP and the FFL showing the most substantial impact on EPPVR, highlighting dataset-specific component contributions.

### 5.2. Parameter Analysis

**Figure 9:**
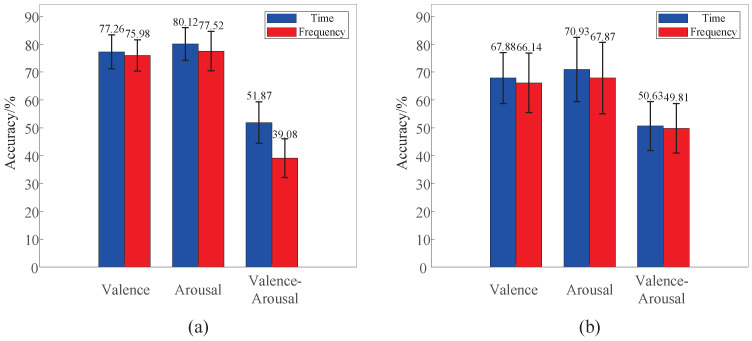
Performance comparison of TSCL-LwF on time-domain and frequency-domain EEG signals. (**a**) DEAP dataset. (**b**) EPPVR dataset.

In existing EEG-based emotion recognition research, frequency-domain features have been widely employed due to their ability to represent band-specific EEG activity. Therefore, we compare the performance of TSCL-LwF using time-domain and frequency-domain EEG signals. As shown in Figure 9a, on the DEAP dataset, time-domain signals consistently outperform frequency-domain signals in valence, arousal, and valence–arousal classification, with accuracy improvements of 1.28%, 2.60%, and 12.79%, respectively. A similar trend is observed on the EPPVR dataset, as illustrated in Figure 9b. This performance advantage can be attributed to differences in feature representation and latent space structure. Time-domain EEG signals preserve waveform morphology, phase information, and cross-channel temporal alignment, which are largely discarded by frequency-domain transformations that primarily emphasize spectral magnitude statistics. These characteristics align well with the multi-scale convolutional modules in TSCL-LwF, enabling the extraction of more discriminative feature representations for emotion recognition. Moreover, time-domain inputs enable the model to learn latent representations with higher intra-class compactness and inter-class separability. As a result, time-domain EEG signals are more suitable for the TSCL-LwF model in emotion recognition tasks.

### 5.3. Influence of Incremental Dataset Size

**Figure 10:**
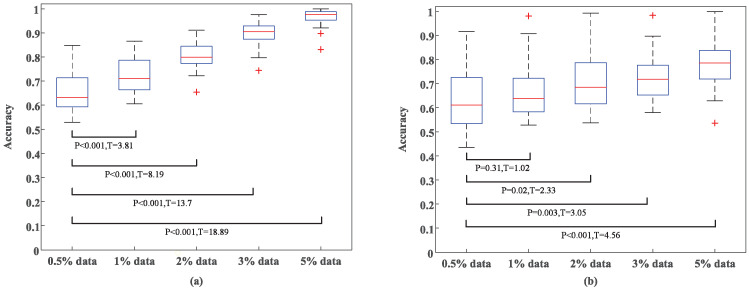
Experimental results of arousal classification with different incremental training data sizes. (**a**) DEAP dataset. (**b**) EPPVR dataset.

To validate the classification performance of TSCL-LwF with different incremental training data sizes, we designed a progressive experiment. The incremental dataset size was set to 0.5%, 1%, 2%, 3%, and 5% of the target domain data. The 0.5% incremental dataset was selected as the baseline as it represents the most data-constrained scenario. Moreover, this setup allows for a more distinct demonstration of the positive impact of data volume expansion on model performance. Figure 10 shows the experimental results of arousal classification trained using different amounts of target domain data. As presented in Figure 10a, on the DEAP dataset, when only 1% of target domain data is used, accuracy exceeds 70%, demonstrating strong adaptation to small samples. When the model is exposed to 3% of target domain data, accuracy surpasses 88.4%, and with 5% of target domain data, it exceeds 96.8%. These results indicate that as the sample size increases, the model effectively learns general features of sparse-channel EEG signals, thus enabling efficient emotion classification. Furthermore, statistical tests with a significance level of p< 0.001 confirm that the accuracy improvement with small amounts of incremental data is not attributable to random fluctuations or extreme values. Figure 10b illustrates that the classification accuracy of the model on the EPPVR dataset also improves with an increase in target domain training samples. However, the accuracy improvement on DEAP is more significant than that on EPPVR as incremental training samples increase. This could be due to the DEAP dataset having a larger incremental dataset than EPPVR, which helps the model learn more EEG characteristics from new subjects, leading to better recognition accuracy.

In this work, a 2% incremental dataset was chosen, and this proportion not only fully adapts to the trial scale of different datasets, but also underwent rigorous verification through statistical significance analysis. Specifically, on DEAP, 2400 samples were extracted from 40 video-elicited trials via time window sliding (with a window length of 1 s and no overlap). The 2% ratio corresponds to 48 samples, which comprehensively cover the features of all videos, and achieves 80.12% accuracy for arousal classification (see Figure 10). This also avoids over-reliance on large volumes of labeled samples, and statistical significance analysis (p<0.001, T = 8.19) confirms that the performance improvement achieved is reliable. For the EPPVR dataset, the 14 original trials were preprocessed into 840 samples. The 2% ratio corresponds to 17 samples, which reduces computational burden while preserving feature discriminability. Notably, only when the incremental dataset size is greater than 2% do all accuracy improvements have statistical significance (p< 0.05). Although using a larger incremental dataset proportion would yield higher recognition accuracy, it leads to pronounced recognition instability. Furthermore, it leads to decreased labeling efficiency and increased computational costs. In summary, these considerations confirm that the 2% incremental dataset proportion strikes an optimal balance among adaptability of the number of trials in the dataset, classification performance, stability, and resource efficiency, making it the most suitable choice for the TSCL-LwF model in this study.

### 5.4. Visualization

**Figure 11:**
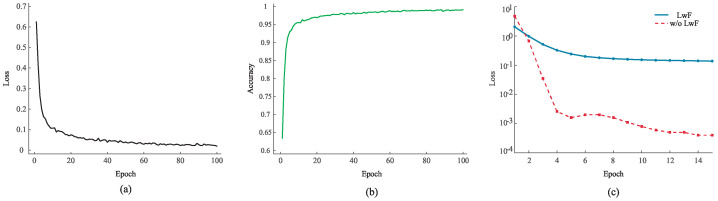
(**a**) The loss curve of the pre-training phase. (**b**) The accuracy curve of the pre-training phase. (**c**) The loss curve of the incremental learning phase.

The training process of the proposed TSCL-LwF on different phases is reported in Figure 11, where the curves were randomly selected from subjects to ensure the representativeness of the experimental results. From Figure 11a, the training loss decreases rapidly within the initial 20 epochs, and then gradually stabilizes with only slight fluctuations. This trend indicates that the model can converge quickly during pre-training, without obvious oscillation or divergence. From Figure 11b, the accuracy can be seen to increase rapidly in the early stage and stabilize at a high level after 20 epochs, with no significant fluctuations or declines. This demonstrates that the model effectively captures related features during learning.

Figure 11c illustrates the dynamic process of LwF and ‘w/o LwF’ during the incremental learning phase. Both models initially show a rapid drop in training loss, indicating that basic feature learning is in progress. Subsequently, the loss of the LwF tends to stabilize at a low level, while the loss of the ‘w/o LwF’ model continues to decrease, but with greater volatility. This reflects that LwF effectively suppresses the interference of new subject learning on old knowledge during incremental training, thus maintaining the stability of model performance across domain stages. Furthermore, the continuously decreasing loss of the ‘w/o LwF’ model may imply overfitting to new data. In contrast, the loss curve of LwF remains stable, which proves that it dynamically balances the learning of new knowledge and the retention of old knowledge during incremental learning, rather than just affecting the final results.

### 5.5. Limitations and Future Research

In this work, we propose a cross-subject emotion recognition model (TSCL-LwF) combining a multi-scale convolutional network (TSCL) and an incremental learning strategy with LwF. Experiments on both the publicly available DEAP dataset and the self-collected EPPVR dataset were conducted to confirm the validity of the proposed model in cross-subject emotion recognition. Compared to methods that rely on multi-channel EEG signals, our proposed method based on a sparse-channel EEG reduces costs in data acquisition and preprocessing. The construction of the TSCL enhances the capability of feature extraction and removes the need for complex feature engineering. Furthermore, the incorporation of the incremental learning strategy with LwF improves cross-subject emotion recognition accuracy and generalization ability, which are especially valuable in applications.

However, this work has several limitations. Firstly, two datasets are used in this study to validate the performance of the model, but there are fewer subjects in the datasets. While the statistical evaluation proves the validity of the proposed model, the limited dataset size might potentially result in high uncertainty. Thus, we will attempt to recruit more subjects to further evaluate the model. Secondly, this work only focuses on two EEG emotion dimensions. Considering the complexity and diversity of human emotions, future research should incorporate more emotion dimensions for a comprehensive analysis.

## 6. Conclusions

In this paper, we propose a cross-subject emotion recognition model (TSCL-LwF) based on sparse-channel EEG to address the practical challenges of wearable emotion recognition. To achieve this objective, a multi-scale convolutional network is designed to extract representative features from sparse-channel prefrontal EEG, while a Learning without Forgetting (LwF) strategy enables efficient subject-incremental adaptation utilizing only a limited set of labeled target domain data. Experimental results on the DEAP and EPPVR datasets demonstrate that the proposed method consistently outperforms existing methods under sparse-channel settings, validating the effectiveness of the proposed design in cross-subject emotion recognition tasks. These findings indicate that TSCL-LwF provides a practical and extensible solution for sparse-channel EEG emotion recognition. Future work will focus on deploying the proposed model on wearable devices to support real-time emotion recognition.

## Figures and Tables

**Table 1:** Relevant details about the datasets.

Factor	DEAP	EPPVR
Subjects	32	30
Trials	40	14
Trial length	63 s (3 s baselines)	70 s (10 s baselines)
Stimuli	Music videos	VR scenes
Dimensions	Arousal, valence	Arousal, valence
EEG equipment	Biosemi Active Two	${\mathrm{eego}}^{TM}$ mylab
Culture	UK	China
Participant ages	19–37	20–26

**Table 2:** Parameter settings.

TSCL-LwF Model Parameters	Values
Batch size	128
Initial learning rate	0.001
Dropout	0.5
Optimizer	Adam
Pre-training phase epochs $N_{s}$	100
Incremental learning with LwF phase epochs $N_{t}$	15
Relaxation parameter λ of LwF	0.1
Temperature coefficient *T* of LwF	2
Random seed	42

**Table 3:** Architecture of the TSCL model.

Layer	Details	Input	Output
MTCL	3-Scale Conv2d,LeakyReLU,AvgPool2d(1,8)	[1,4,128]	[128,4,34]
	Kernel=128@(1,64)/(1,32)/(1,16)		
	Feature Concatenation,BN2d		
MSCL	2-Scale Conv2d,LeakyReLU,AvgPool2d(1,2)	[128,4,34]	[15,3,17]
	Kernel=15@(2,1)/(4,1)		
	Feature Concatenation,BN2d		
FFL1	Separable Conv2d,BN2d,ReLU,MaxPool2d(1,4)	[15,3,17]	[1,15]
	Kernel=15@(3,1)		
	Reshape		
FFL2-4	3-Layer Separable Conv1d,BN1d,ReLU,MaxPool1d(2)	[1,15]	[128,3]
	Kernel=32/64/128@(3,1)		
DCL	3-Layer Linear,BN1d,ReLU,Dropout	[128,3]	[2]

**Table 4:** Comparison of accuracy and F-Score (mean ± standard deviation (STD)) for different models on the DEAP dataset, with bold data indicating the highest classification performance among the models.

Method	Valence		Arousal		Valence–Arousal	
	**Accuracy (%)**	**F-Score (%)**	**Accuracy (%)**	**F-Score (%)**	**Accuracy (%)**	**F-Score (%)**
ShallowConvNet [36]	51.78±6.3 ***	38.10±4.44 ***	51.04±10.71 ***	36.91±7.18 ***	27.27±7.81 ***	15.07±4.85 ***
DGCNN [14]	49.56±4.72 ***	37.96±3.95 ***	50.45±7.02 ***	38.27±5.08 ***	27.37±6.53 ***	14.93±5.43 ***
CLISA [24]	55.07±9.3 ***	46.43±10.53 ***	57.57±15.93 ***	47.47±16.3 ***	40.01±10.83 ***	22.76±9.32 ***
TSception [37]	49.23±6.79 ***	36.56±5.54 ***	51.12±8.55 ***	38.22±5.92 ***	24.52±6.24 ***	13.13±3.39 ***
LGGNet [38]	51.55±6.68 ***	38.17±5.19 ***	51.74±10.18 ***	38.60±8.28 ***	28.7±7.09 ***	15.57±37.3 ***
MACTN [39]	49.19±6.38 ***	43.17±10.17 ***	51.15±8.02 ***	48.74±13.35 ***	25.7±5.8 ***	14.18±3.84 ***
EmT [25]	51.76±7.73 ***	38.31±5.55 ***	51.26±11.1 ***	37.5±8.98 ***	28.96±8.02 ***	17.45±5.94 ***
TSCL-LwF	**77.26 ± 5.01**	**76.25 ± 4.74**	**80.12 ± 5.87**	**76.02 ± 5.54**	**51.87 ± 7.47**	**42.06 ± 5.21**

*p*-value of the improvement of TSCL-LwF over the model: *** indicated (*p*< 0.001).

**Table 5:** Comparison of accuracy and F-Score (mean ± standard deviation (STD)) for different models on the EPPVR dataset, with bold data indicating the highest classification performance among the models.

Method	Valence		Arousal		Valence–Arousal	
	**Accuracy (%)**	**F-Score (%)**	**Accuracy (%)**	**F-Score (%)**	**Accuracy (%)**	**F-Score (%)**
ShallowConvNet [36]	58.41±12.78 **	40.04±7.31 ***	47.38±11.96 ***	37.85±8.23 ***	28.87±11.01 ***	15.83±4.91 ***
DGCNN [14]	59.99±11.07 **	41.06±6.11 ***	47.51±10.9 ***	38.65±9.22 ***	28.65±9.77 ***	15.40±5.05 ***
CLISA [24]	63.6±15.32	53.54±17.22 *	49.46±14.24 ***	39.22±11.71 ***	34.75±17.32 ***	21.76±10.02 ***
TSception [37]	56.10±8.77 ***	41.07±4.35 ***	46.84±7.51 ***	39.17±6.89 ***	27.65±15.84 ***	16.28±11.86 ***
LGGNet [38]	58.97±11.46 **	39.03±5.82 ***	48.58±10.13 ***	39.98±9.15 ***	28.43±9.25 ***	15.46±4.61 ***
MACTN [39]	59.26±13.44 *	41.04±8.91 ***	49.69±11.25 ***	39.18±9.36 ***	29.08±9.46 ***	16.2±4.47 ***
EmT [25]	56.89±10.47 ***	38.79±5.12 ***	46.9±9.53 ***	37.84±6.71 ***	25.85±7.67 ***	13.87±3.3 ***
TSCL-LwF	**67.88 ± 9.14**	**62.02 ± 8.04**	**70.93 ± 11.51**	**61.71 ± 7.42**	**50.63 ± 8.79**	**39.76 ± 8.53**

*p*-value of the improvement of TSCL-LwF over the model: *** indicates (*p* < 0.001), ** indicates (*p* < 0.01), * indicates (*p* < 0.05).

**Table 6:** Results of ablation experiments of TSCL-LwF in arousal for the DEAP and EPPVR datasets.

Method	DEAP		EPPVR	
	**ACC (%)**	**F-Score (%)**	**ACC (%)**	**F-Score (%)**
w/o MTCL	78.65 ± 6.1	74.01 ± 4.64	68.74 ± 13.71	59.91 ± 9.28
w/o MSCL	67.88 ± 8.22	60.72 ± 5.95	62.29 ± 19.02	44.93 ± 17.08
w/o FFL	75.07 ± 9.3	66.43 ± 10.53	59.57 ± 16.93	43.47 ± 11.37
w/o LwF	74.23 ± 4.95	71.56 ± 5.34	61.12 ± 7.15	56.22 ± 9.02
TSCL-LwF	80.12±5.87	76.02±5.54	70.93±11.51	61.71±7.42

## Data Availability

The original code and data presented in the study are openly available at https://github.com/19165517212/TSCL-LwF (accessed on 6 January 2026).
